# Machine Learning Allowed Interpreting Toxicity of a Fe-Doped CuO NM Library Large Data Set—An Environmental In Vivo Case Study

**DOI:** 10.1021/acsami.4c07153

**Published:** 2024-08-01

**Authors:** Janeck J. Scott-Fordsmand, Susana I.L. Gomes, Suman Pokhrel, Lutz Mädler, Matteo Fasano, Pietro Asinari, Kaido Tämm, Jaak Jänes, Mónica J.B. Amorim

**Affiliations:** † Department of Ecoscience, Aarhus University, C.F. Mo̷llers Alle 4, DK-8000 Aarhus, Denmark; ‡ Department of Biology & CESAM, University of Aveiro, 3810-193 Aveiro, Portugal; § Department of Production Engineering, University of Bremen, Badgasteiner Str. 1, 28359 Bremen, Germany; ∥ Leibniz Institute for Materials Engineering IWT, Badgasteiner Str. 3, 28359 Bremen, Germany; ⊥ Department of Energy, Politecnico di Torino, Corso Duca degli Abruzzi 24, Torino 10129, Italy; # INRIM, Istituto Nazionale di Ricerca Metrologica, Strada delle Cacce 91, Torino 10135, Italy; ∇ Institute of Chemistry, University of Tartu, Ravila 14a, Tartu 50411, Estonia

**Keywords:** machine learning, soil, ecotoxicology, safer and sustainable-by-design (SSbD), advanced materials

## Abstract

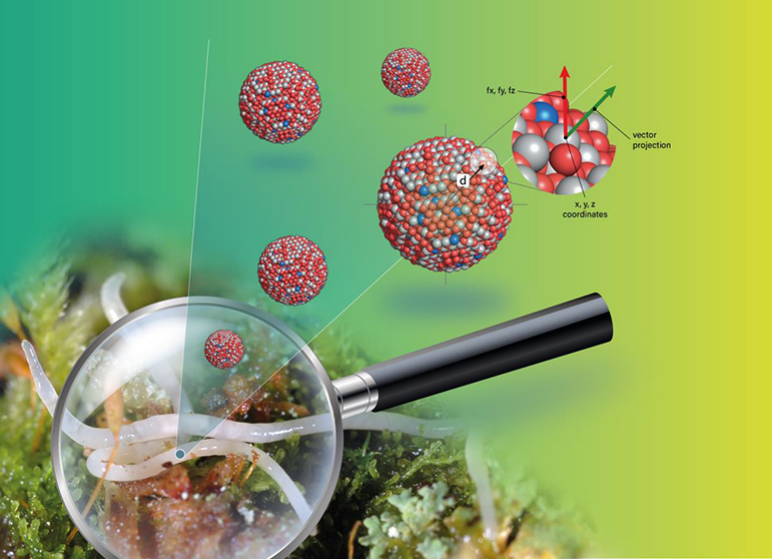

The wide variation of nanomaterial (NM) characters (size, shape, and properties) and the related impacts on living organisms make it virtually impossible to assess their safety; the need for modeling has been urged for long. We here investigate the custom-designed 1–10% Fe-doped CuO NM library. Effects were assessed using the soil ecotoxicology model *Enchytraeus crypticus* (Oligochaeta) in the standard 21 days plus its extension (49 days). Results showed that 10%Fe-CuO was the most toxic (21 days reproduction EC50 = 650 mg NM/kg soil) and Fe_3_O_4_ NM was the least toxic (no effects up to 3200 mg NM/kg soil). All other NMs caused similar effects to *E. crypticus* (21 days reproduction EC50 ranging from 875 to 1923 mg NM/kg soil, with overlapping confidence intervals). Aiming to identify the key NM characteristics responsible for the toxicity, machine learning (ML) modeling was used to analyze the large data set [9 NMs, 68 descriptors, 6 concentrations, 2 exposure times (21 and 49 days), 2 endpoints (survival and reproduction)]. ML allowed us to separate experimental related parameters (e.g., zeta potential) from particle-specific descriptors (e.g., force vectors) for the best identification of important descriptors. We observed that concentration-dependent descriptors (environmental parameters, e.g., zeta potential) were the most important under standard test duration (21 day) but not for longer exposure (closer representation of real-world conditions). In the longer exposure (49 days), the particle-specific descriptors were more important than the concentration-dependent parameters. The longer-term exposure showed that the steepness of the concentration–response decreased with an increased Fe content in the NMs. Longer-term exposure should be a requirement in the hazard assessment of NMs in addition to the standard in OECD guidelines for chemicals. The progress toward ML analysis is desirable given its need for such large data sets and significant power to link NM descriptors to effects in animals. This is beyond the current univariate and concentration–response modeling analysis.

Nanomaterials (NMs) enter the market at an unprecedent pace, as never seen before for any class of chemicals.^1−3^ At this pace, it is difficult to timely evaluate their risks, let alone that it is virtually impossible to assess all variations of existing NMs. As any other material, NMs can pose serious threats to human health^4^ and to the environment.^5−7^ The (eco)toxicity of NMs, when evaluated, is mostly done for one or a few NMs at a time and based on one or a few species/cell lines. This is well-known to be a rather time-consuming and low-efficient process. Hence, it is important to progress toward a modeling-based approach and develop good predictor-based case studies to support the transition. Data modeling can be done via different methods, e.g., quantitative structure–activity relationship (QSAR) analysis and machine learning (ML), among others, where many material features can be analyzed at the same time and used to predict toxicity.^8−11^

In recent years, ML algorithms have emerged as powerful tools in the field of nanotoxicology, offering a promising avenue to predict and analyze the toxicity of NMs more efficiently and accurately.^12^ ML enables toxicity prediction by training models on data sets of NMs with known outcomes, making them well-suited for the multifaceted nature of NM toxicity.^13^ A diverse range of ML attempts have been made in the context of nanotoxicology, contributing to our understanding of the relationships between NM properties and their potential effects on living systems.^14,15^

ML models can utilize physicochemical and structural properties, such as particle size, surface charge, and composition, to estimate the likelihood of adverse effects.^9^ Additionally, ML techniques have been exploited for establishing QSAR, shedding light on the molecular mechanisms underlying toxicity and guiding safer-by-design NM.^16^ Furthermore, ML can address challenges in data imputation and integration, facilitating comprehensive and reliable toxicological analyses.^17^ ML has been also used to model adverse outcome pathways, identify critical pathways through which NMs exert adverse effects, and explore clustering and pattern recognition to identify distinct toxicity pathways and correlations with physicochemical properties.^18,19^ ML also has the potential to enable cross-species toxicity extrapolation, bridging knowledge gaps between different organisms and facilitating risk assessment for humans and other species.^20^ Last, feature selection and importance analysis can be employed to identify key descriptors influencing NM toxicity and enhance model interpretability.^21^ One way to sort out the descriptors of toxicity of NMs from a data set using ML is to identify the descriptors closely associated with biological endpoints and then determine the strength of association of the identified descriptors with the respective biological endpoint.^22^ Therefore, ML holds significant potential in nanosafety, but addressing challenges is crucial to ensure reliable and applicable models.^23^ A major challenge is the availability and quality of toxicological data for NMs, where insufficient or inconsistent data can hinder accurate predictions and introduce biases. Interpreting ML predictions is another critical issue as many algorithms operate as black boxes: ensuring model interpretability is essential to gain mechanistic insights into NM toxicity and building trust in predictions. Moreover, the complex interactions between NMs and biological systems lead to nonlinear toxicity responses, challenging traditional linear models. To achieve broader model applicability across different types of NMs and exposure scenarios remains a challenge. Careful consideration of data set composition and feature representation during model training is essential to enhance generalization.^24^

Studies performed under comparable conditions and including many NMs are necessary to derive valid models, but these studies are seldom available, although they are particularly relevant. To pursue such a quest, libraries (sets of custom-designed NMs with varying characters, e.g., size, while keeping other variables constant) can be used as they provide large sets of character-dependent descriptors that can be used for modeling toxicity when tested under uniform conditions. Data modeling anchored to NM libraries testing has previously allowed the identification of specific NM properties that trigger toxicity^15,25−27^ and also identified the biological mechanisms of response.^28^ Hence, such a combination of uniform experimental data with computational modeling can lead to the reduction in numbers of tests required for hazard assessment.

In the present study, we investigate the bioactivity of an Fe-doped CuO NM library based on in vivo toxicity assays. Effects were assessed using the soil ecotoxicology model *Enchytraeus crypticus* (Oligochaeta) based on the Organization for Economic Co-operation and Development (OECD) standard 21 days enchytraeid reproduction test (ERT)^29^ and a standard extension up to 49 days. ML modeling was used to analyze the large data set (9_[NMs]_*68_[descriptors]_*6_[concentrations]_*2_[exposure times: 21, 49 days]_*2_[biological endpoints: survival, reproduction]_). Enchytraeids are widely distributed in soils worldwide, where they contribute to improving the soil structure and organic matter decomposition. Further, they are the most important organisms in many soil habitats dominant in biomass or abundance.^30^ The Fe-CuO NM library studied was a custom-designed combinatorial library in which CuO was doped with 1–10% Fe in a flame spray pyrolysis reactor.^27^ The library (pristine particles) was fully characterized by X-ray diffraction (XRD), Brunauer–Emmett–Teller (BET) method, Raman spectroscopy, transmission electron microscopy [TEM: high-resolution TEM and energy filtered TEM], and electron energy loss spectroscopy^27^ in addition to dynamic light scattering (DLS) and zeta potential.

Fe doping constitutes a safer-by-design alternative to the toxic CuO NM, as demonstrated by a progressive decrease in cytotoxicity to BEAS-2B and THP-1 cells as well as an incremental decrease in the rate of hatching interference in zebrafish embryos.^27^ A decrease in dissolution in the aqueous test media (and Cu^+/2+^ release) with increase in Fe% was associated with the decrease in toxicity.^27^ However, no such information exists for the soil compartment, and the toxicity to soil organisms is unknown. The aims of the present study are to assess the environmental toxicity of a fully characterized Fe-doped CuO NM library and to identify the key NM characteristics responsible for the toxicity, the latter using ML modeling to analyze the large data set obtained.

## Results

### Material Characterization

**Figure 1:**
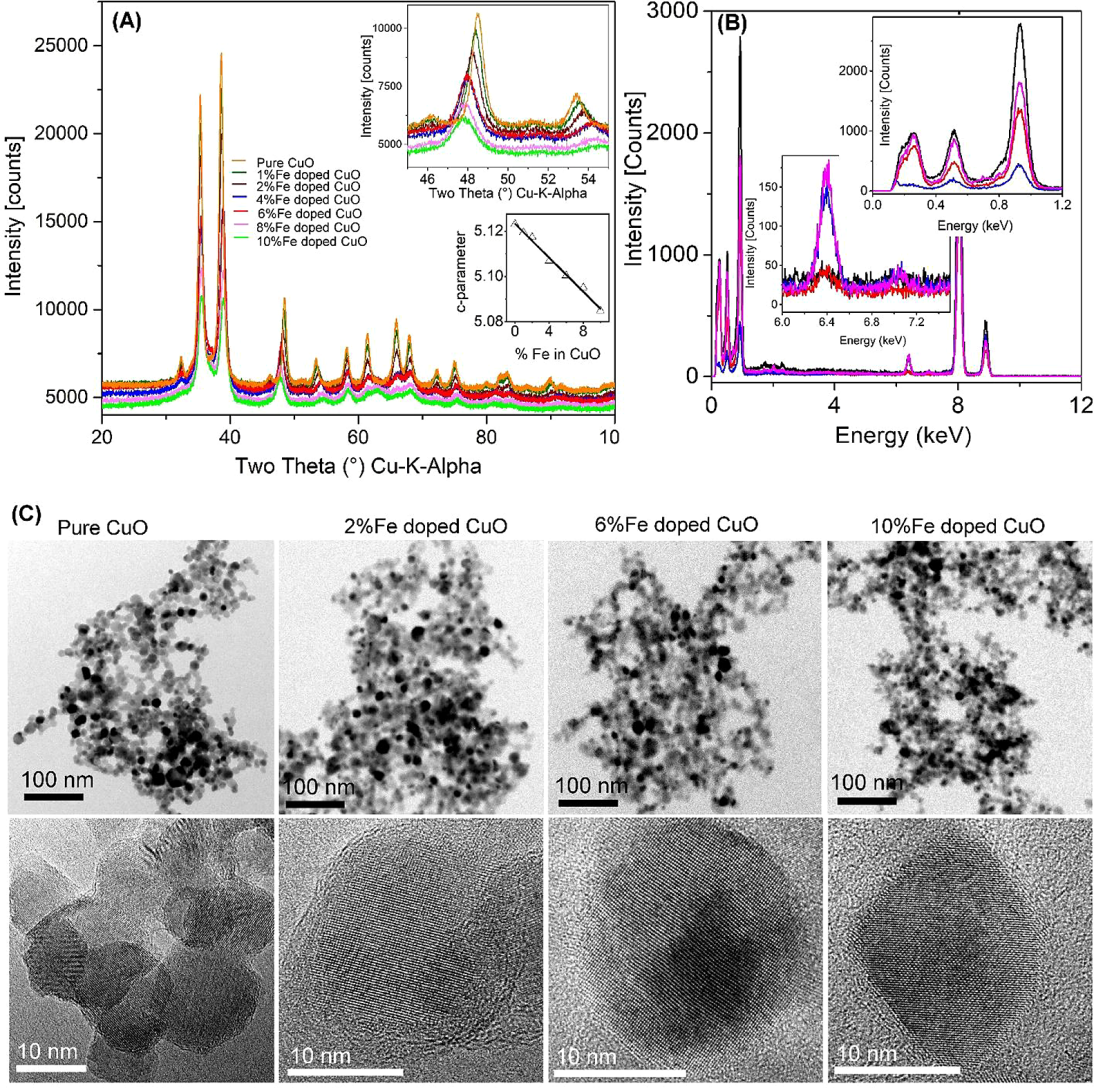
Physicochemical characterization of pure and Fe-doped CuO nanoparticles. (A) XRD patterns of Fe-doped CuO homologous series. The data show that the patterns are slightly shifted with doping (figure inset, top) corresponding to the linear decrease of the *c*-parameter of the CuO unit cell (figure inset, bottom). (B) EDX analysis of the dry powder. The particle composition reasonably agrees with the initial amounts of Cu and Fe in the feed solution prepared for combustion. The C-signal observed is due to C in the TEM grid. (C) Low-resolution (top row) and high-resolution (bottom row) of pure and doped particles. The data show that the agglomerated particles are highly crystalline.

The flame-made homologous series of Fe-doped CuO particles were analyzed using advanced characterization techniques.^31^ The high intensity X-ray diffraction patterns of pure and Fe-doped CuO indicate highly crystalline particles (Figure 1A).

**Figure 2:**
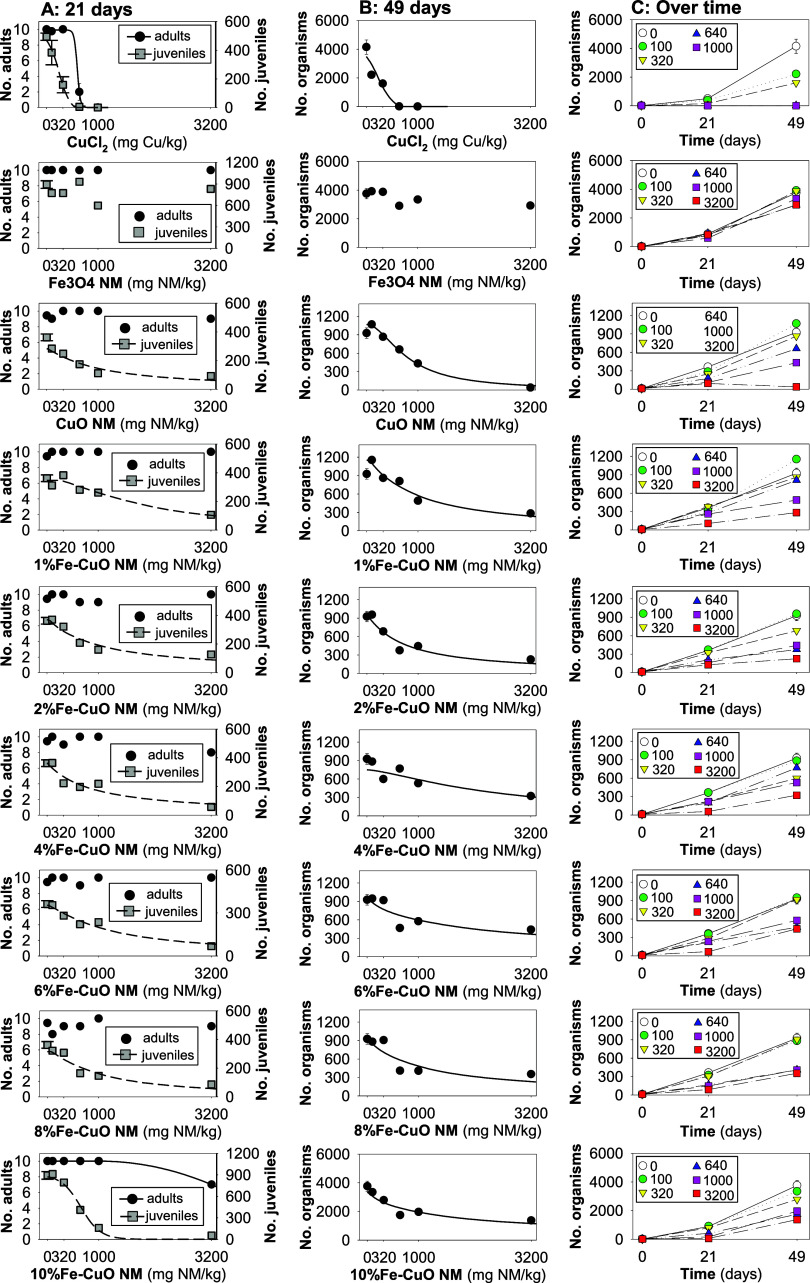
Results in terms of survival and reproduction of *Enchytraeus crypticus* exposed to CuCl_2_ and 8 NMs: Fe_3_O_4_, pure CuO, and 1, 2, 4, 6, 8 and 10% Fe-doped CuO in LUFA 2.2 soil during (A) 21 days, (B) 49 days, and (C) over 0, 21, and 49 days. For (A) and (B), the lines represent the model fit to data.

**Figure 3:**
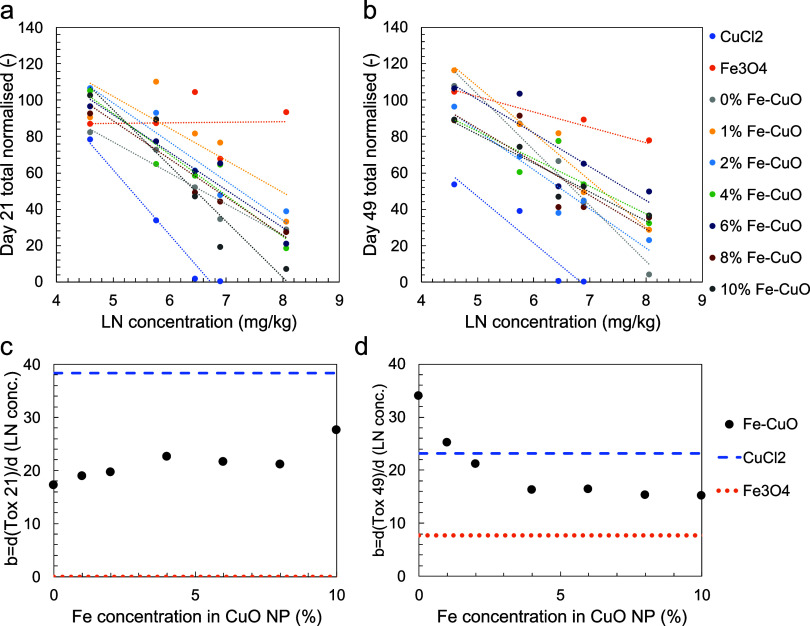
Best fit of the biological response (*y*) to Fe-doped CuO NMs after (a) 21 days and (b) 49 days of exposure with respect to the given concentration (*c*). Colored dots correspond to experimental measurements; dashed lines (with the same color as the corresponding particle or substance tested) correspond to the best fitting function in the form *y* = −*b* ln *c* + *b*_0_. The best fitted value of the *b* parameter, namely, the derivative of the biological end point with respect to the natural logarithm of the concentration, is shown for (c) 21 days and (d) 49 days of exposure as a function of Fe concentration in the tested CuO NMs. See Tables S3 and S4 for a detailed list of the fitting parameters and accuracy.

The BET primary particle sizes (*d*_BET_) of pure and Fe-doped CuO were found to be in the range of 10–14 nm (see Table 1) indicating ultrafine nature of the particles (for full details, see (27)). The powder XRD patterns of pure and Fe-doped nanoparticles were Rietveld refined (ICSD 69757, space group C1C1). The X-ray data showed (1) a decrease in the X-ray intensity (Figure 1A), (2) a slight peak shift with Fe doping (upper inset in Figure 1A), and (3) high crystallinity with crystallite sizes in the range of 10–12 nm (Table 1).

**Table 1:** Specific Surface Area (SSA), Primary Particle Size (dBET), and Crystallite Size (dXRD) for Pure and Fe-doped CuO NMs^27^

particles	SSA (m^2^/g)	*d*_BET_ (nm)	*d*_XRD_ (nm)
pure CuO	80.9 (±2.5)	11.8 (±1.3)	9.4 (±0.1)
1% Fe-doped CuO	79.2 (±3.2)	12.0 (±1.5)	11.9 (±0.4)
2% Fe-doped CuO	77.6 (±1.8)	12.3 (±1.2)	9.2 (±0.1)
4% Fe-doped CuO	89.6 (±4.2)	10.7 (±1.8)	10.5 (±0.1)
6% Fe-doped CuO	92.9 (±3.6)	10.3 (±1.6)	10.8 (±0.1)
8% Fe-doped CuO	93.6 (±4.5)	10.3 (±1.9)	9.8 (±0.4)
10% Fe-doped CuO	90.4 (±1.2)	10.7 (±1.0)	9.6 (±0.9)
pure Fe_3_O_4_	80.4	14.5	-

While the refinement of XRD patterns of Fe-doped CuO was performed with the pure CuO cif file, reasonable fitting of the refined patterns indicates Fe incorporation going beyond the known solubility limit. To verify high doping possibility, the *c*-parameters of pure and Fe-doped CuO were plotted against Fe content. The almost perfect linear behavior for all doping agrees with Vegards rule (see lower inset in Figure 1A). The pure and/or doped particle composition was investigated using energy-dispersive X-ray spectroscopy (EDX), and the data showed precise amounts of Fe and Cu that were added in the feed solution before flame combustion (Figure 1B). TEM of pure and Fe-doped CuO homologous series shows spherical particles and very similar morphology with a particle distribution window of 5–20 nm (Figure 1C, upper and lower columns). The images of the particles confirm the high crystallinity observed in the XRD (for detail characterization of the particles, please see refs (27, 32, and 33)). DLS results (Table S1) evidenced the high degree of agglomeration of the particles when dispersed in water, but that was not a clear concentration-dependent pattern. The zeta potential results corroborate the high instability of the system, i.e., values ranging from −14 to 10 mV.

### Material Modeling

The calculated all-atom full particle nanodescriptors (Table S1) describe the core and surface regions of the NPs. These descriptors cover the total number of atoms (both Cu and Fe) and are based on the chemical composition, potential energy, lattice energy, topology, size, and force vectors. Constitutional descriptors are the counts of atoms of different identities and/or location. Potential energy descriptors are derived from the force-field calculations corresponding to the arithmetic means of the potential energies for specific atom types and/or locations in the NM. Lattice energies are based on the same potential energies but presented as metal oxide nominal units (MxOy) and describe the energy needed to rip away the said unit from the nanoparticle surface. All potential energy-related descriptors are presented in units of eV. The coordination number of atoms is defined as the count of the neighboring atoms which lie inside the radius *R*: *R* = 1.2 × (*R*_M_ + *R*_O_), where *R*_M_ and *R*_O_ are the ionic radii of metal and oxygen ions, respectively.

### Materials Bioactivity (Effects on Survival and Reproduction)

For the standard 21-day ERT, the validity criteria were fulfilled as within the standard OECD test guideline,^29^ i.e., in controls, adult mortality <20%, and the number of juveniles >50 per replicate, with a coefficient of variation <50%.

The results showed that in the standard 21-day exposure, CuCl_2_ and 10%Fe-CuO NM caused a concentration-dependent reduction on survival (CuCl_2_ being the most toxic), while the other tested NMs did not cause effects (Figure 2A). In terms of reproduction, there was a concentration-dependent decrease in reproduction for all of the tested materials, except Fe_3_O_4_ NM (Figure 2A).

CuCl_2_ was the most toxic (EC50 = 244 mg Cu/kg soil), followed by 10%Fe-CuO NM (EC50 = 650 mg NM/kg soil). For all other NMs, the ECx determined are similar (with overlapping confidence intervals), as can be seen in Table S2.

In the prolonged exposure (49 days, standard extension), the toxicity of 10% Fe-decreased to the same level as the other NMs (EC50 with overlapping intervals), which resembles the effects observed on day 21 in terms of reproduction (Figure 2 and Table S2).

### ML Data Analysis

The multistep data analysis method was utilized to identify the descriptors responsible for the biological response of Fe-doped CuO NMs from a list of experimental and modeling variables potentially involved in the toxicological mechanisms. Starting with an initial set of *N* = 68 variables (*x*_1_, *x_2_,* ..., *x*_68_), the data cleaning process reduced the number to *N* = 67. In this preliminary cleaning process, we removed the XRD (dXRD) data from the analysis as this variable had missing values for some configurations of the nanomaterials (see Table S8). The presence of incomplete data for dXRD could introduce biases and inconsistencies in the ML model, potentially affecting the reliability and accuracy of the results. Therefore, to maintain the integrity of the analysis, it was essential to exclude this variable, ensuring that all potential descriptors used in the model had complete and consistent data across all configurations.

The 21- and 49-days biological responses were then fitted as a function of concentration of the tested NMs (Fe-doped CuO, Fe_3_O_4_) or substance (CuCl_2_) as reported in Figure 3a,b, respectively.

The observed high fitting accuracy (*R*^2^ between 0.61 and 0.97, cf. Tables S3 and S4) demonstrates a common logarithmic decrease of the biological response with concentration. The only notable exception was the test with Fe_3_O_4_ NMs, which showed a concentration-independent biological response after 21 days that eventually became dependent on the concentration after 49 days. The best fitted values of the *b* parameter, namely, the derivative of the biological end point with respect to the natural logarithm of the concentration (i.e., the steepness of the response curve), is shown for 21-days and 49-days exposure in Figure 3c,d, respectively, as a function of Fe concentration in the tested CuO NMs. In the case of 21-days exposure, the highest *b* value (i.e., the steepest decrease in biological response with concentration increase) was found for CuCl_2_, while the lowest was found for Fe_3_O_4_ NMs. Fe-doped CuO NMs showed intermediate values of *b* with respect to CuCl_2_ and Fe_3_O_4_, without any statistically relevant change with the Fe concentration. Prolonging the exposure up to 49 days led to a clear relationship between *b* and Fe concentration in the Fe-doped CuO NMs, with higher *b* values at low Fe concentrations (the highest value was found for the CuO NM, i.e., 0% Fe concentration). The *b* value of Fe-doped CuO NMs eventually decreased to a constant value at higher Fe concentrations. Furthermore, the 49-days exposure also showed a lower *b* value for CuCl_2_ and a higher *b* value for Fe_3_O_4_ with respect to 21-days exposure observations, highlighting that different toxicological mechanisms may be triggered with longer exposure time. Overall, CuCl_2_ appears to be the substance causing the sharpest decrease in the biological response with concentration, followed by Fe-doped CuO and finally Fe_3_O_4_ nanoparticles. Notably, Fe-doped CuO nanoparticles with low Fe doping can overcome the *b* value of CuCl_2_ in the case of 49-days exposure.

The concentration-independent end point *b* eliminates the obvious effect of concentration on the biological response and hence allows for a clearer analysis of chemical-physical effects of the considered nanoparticles on toxicity, which showed a clear impact at least for 49-days exposure. Therefore, concentration-dependent variables were initially removed from the analysis, and the hierarchical clustering algorithm was employed to highlight correlated variables (Figure S2). The algorithm revealed 15 clusters of similar variables, and the clustering accuracy was confirmed by the high values of the cophenetic correlation coefficient and Spearman’s correlation coefficients within each cluster (Figure S3). The representative variable per each cluster was nominated (Table S5), and then, the pruning process was iteratively conducted. The process terminated at the fourth round (Figure S4), and the remaining three variables after pruning (Table S6) were considered significant descriptors of the toxicological mechanism related to Fe-doped CuO NMs. The identified concentration-independent descriptors included a balanced mix of geometrical (nanoparticle diameter), geometrical-environmental (surface specific area of nanoparticles, which may be influenced by the surrounding medium), and chemical (force vector of metallic/oxygen atoms in the nanoparticles, cf. cluster #9 in Table S5) features.

**Figure 4:**
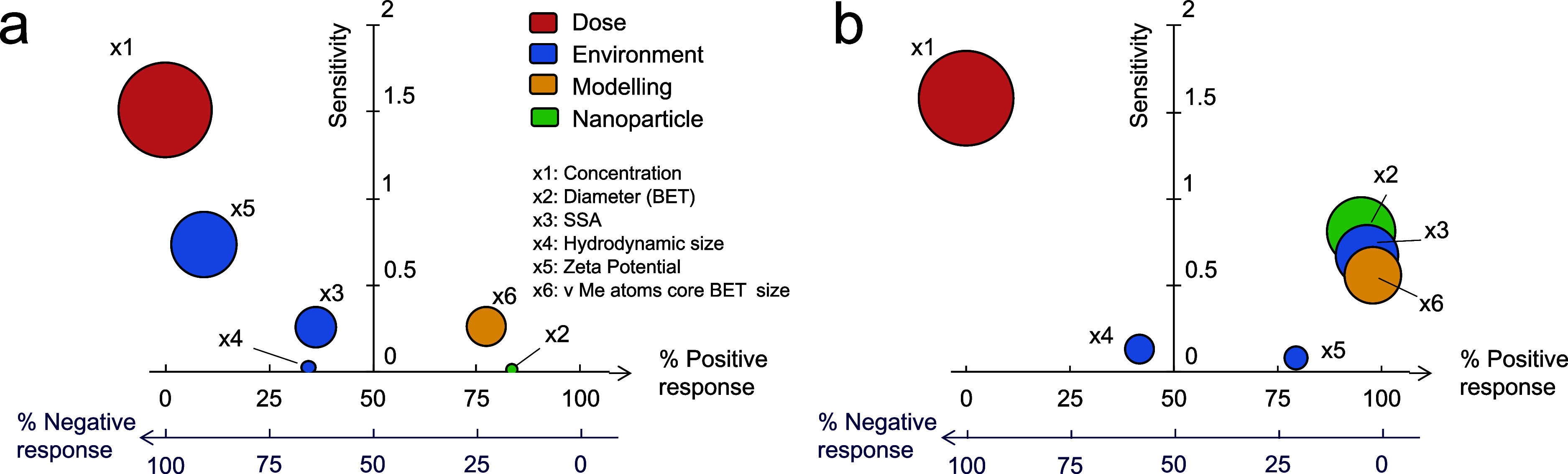
Effect of descriptors on the biological response. The process of variables pruning reveals the presence of six descriptors that significantly influence the biological response of Fe-doped CuO NMs (see Table S7). The sensitivity of the biological response to these descriptors is depicted for (a) 21 days of exposure and (b) 49 days of exposure. In this context, “sensitivity” quantifies the average relative impact of a descriptor within the identified fitting models on the biological response. On the other hand, “% positive response” refers to the likelihood that increasing a descriptor will lead to an increase in the biological parameters (i.e., number of organisms) and vice versa for “% negative response.” For example, a “% positive response = 80% for the *x*_6_ descriptor (21-days exposure)″ means that, considering the explored fitting functions and values of *x*_6_ between the minimum and maximum in the data set, *x*_6_ is positively correlated with the biological response in 80% of the cases. In the remaining 20% of cases, a negative or no correlation is observed instead.

Finally, concentration-dependent variables such as concentration, hydrodynamic size, and zeta potential were added back into the analysis, leading to the final set of descriptors listed in Table S7 that proved to be all uncorrelated between each other (cf. Figure S5). Considering this final list of six descriptors, the symbolic regressor was used to best fit the biological response and highlight its sensitivity on each descriptor for both 21- and 49-days exposure. Figure 4a (21-days exposure) and b (49-days exposure) remarks the obvious evidence that the biological response had the highest sensitivity on the nanoparticle concentration. For the 21-days exposure, relevant descriptors were also, in descending order of sensitivity, zeta potential, force vector of metallic/oxygen atoms in the nanoparticles, and their surface specific area; whereas for the 49-days exposure, they were the diameter of nanoparticles, their surface specific area, and the force vector of metallic/oxygen atoms in the nanoparticles. Such evidence highlighted descriptors significantly affecting both exposure times (i.e., surface specific area of nanoparticles and force vector of metallic/oxygen atoms in the nanoparticles) or not (i.e., hydrodynamic size) and descriptors with different influence on the biological response according to the exposure time. In particular, the zeta potential appeared as a clear parameter of the toxicity mechanisms involved in the first 21 days of exposure; whereas the diameter of nanoparticles showed a significant influence in the 49-days exposure scenario. Such a discrepancy of the second-order effects on the biological response may be a further confirmation of different toxicological mechanisms as time is prolonged. Figure 4a,b provides additional insights into the “% positive response” of descriptors on the biology. This quantity indicates the likelihood of increasing the biological response with an increase in the descriptor. The observed negative correlation between the concentration and biological response aligns with typical findings in the literature. The hydrodynamic size also showed a mostly negative correlation with the biological response, while the nanoparticle diameter and the force vector of their metallic/oxygen atoms were a positive one: in other words, smaller nanoparticles with lower force vector of their metallic/oxygen atoms and higher hydrodynamic size should decrease the biology most. The zeta potential, instead, demonstrated a different response according to the exposure time, switching from a strongly negative (21-days exposure) to a mostly positive (49-days exposure) correlation with the biological response.

In the last extended fitting by the symbolic regressor, the best correlations between the six descriptors listed in Table S7 and the biological response achieved remarkable *R*^2^ values of 0.93 for 21 days of exposure and 0.94 for 49 days of exposure experiments (see Figure S6).

## Discussion

The in vivo toxicity results showed that the biological response of *E. crypticus* to Fe-doped CuO NMs depends on the Fe%, and that the patterns of toxicity observed after the standard exposure test (21 days) were overall maintained after prolonged exposure (49 days).

Based on the standard test (21 days) and based on literature data,^34^ CuCl_2_ was the most toxic in terms of survival and reproduction. The 21-days EC50 determined here (244 mg Cu/kg soil) is in good agreement with that reported previously for *E. crypticus* (28-days EC50 = 179 mg Cu/kg soil), with overlapping confidence intervals.^34^ The effects observed for CuO NM were also in agreement with literature data when compared to a commercial CuO NM (Plasma Chem) of similar primary particle size (ca. 12 nm).^34^ Among the tested materials, the NM with the steepest concentration–response curve was the 10%Fe-CuO (21-days reproduction EC50 = 650 mg NM/kg soil), and the least toxic was Fe_3_O_4_ NM (no effects up to 3200 mg NM/kg soil). All other NMs caused similar effects to *E. crypticus* (21-days reproduction EC50 ranging from 875 to 1923 mg NM/kg soil, with overlapping confidence intervals). A previous study showed that increasing the Fe doping of CuO NM caused a progressive reduction of toxicity to BEAS-2B and THP-1 cells and to zebrafish embryos.^27^ For the cell lines, 6, 8, and 10%Fe showed similar performance in protecting cell viability.^27^ Also, Joshi et al.^35^ showed that DMSA-coated CuO NM was more toxic to C6 Glioma cells than DMSA-coated 10%Fe-CuO NM. Both studies^27,35^ point at the higher dissolution and Cu^2+^ release, from pure CuO NM, in the test media (diverse cell culture media) as the cause for higher toxicity.

For the CuO NM library tested here, the particle dissolution in aqueous solution was insignificant.^27^ A low dissolution pattern was also reported for CuO and 10%Fe-CuO in freshwater, with minimal toxicity, in terms of growth inhibition of the marine algae *Isochrysis galbana* and sea urchin.^36,37^ In this work, the NMs were added to soil as dry powders, and the adding of water to moist the soil would lead to minimal dissolution unlike the results reported by Naatz et al.^27^ The reported data indicated ionic release leading to strong covalent complexation between metal ions (M^z+^) and proteins/amino acids in the cells, subsequent precipitation of the Cu-complex crystals in the cellular fluid, and ROS generation, followed by Tier III biological cascades. The in vivo particle exposure was long term, and the dissolution kinetics of pure and Fe-doped CuO over 250 h was evaluated. The dissolution profile revealed an initial fast burst-like release of copper, followed by a prolonged slow release lasting several weeks. While the particle exposure in the present investigation was based on the aqueous medium, the differences observed between the pure and doped CuO NMs are not related to Cu^2+^ release, instead the Fe above a certain threshold (>6%) might play a role to affect surface activity. Considering that Fe_3_O_4_ NM was not toxic to *E. crypticus*, the higher effects observed for 10%Fe-CuO might result from the interaction between Cu and Fe.^38^ With increasing exposure time (up to 49 days), the toxicity of 10%Fe-CuO NM was reduced to the same level as all other NMs.

Overall, across concentration-dependent variables, the exposure concentration is the most important factor, i.e., in all cases (except Fe_3_O_4_ for 21 days) an increase in concentration leads to a decrease in the number of juveniles (Figure 2). The steepness of the concentration–response curve decreased with the increase in %Fe-doping, but a correlation was observed only after 49 days. It is related to a stronger stability (higher values of the force vector related descriptors) of the particle with lower %Fe. For the 21-day exposure, the zeta potential (range 5–10 mV) is also negatively correlated in most cases with a high sensitivity, whereas this is not the case for the 49-day exposure where zeta potential was mainly positively correlated but less sensitive in the model. The difference may be due to aggregation/agglomeration to the soil matrix being more important with longer exposure time. For the 49-day exposure, also the hydrodynamic size was, in less cases, related to the biology and not sensitive in the model, possibly again due to the aggregation/agglomeration over time. In other words, the importance of zeta potential in the standard test duration (21 days) can be attributed to its effect on the initial agglomeration and sedimentation behavior of nanoparticles. During the early phase of exposure, nanoparticles with higher absolute zeta potential values (either positive or negative) tend to repel each other, resulting in higher dispersion in the soil matrix. This increased dispersion enhances the bioavailability of nanoparticles, thereby increasing their potential for toxic interactions with soil organisms such as *Enchytraeus crypticus*. Conversely, nanoparticles with low absolute zeta potential values are more prone to agglomeration, which reduces their mobility and bioavailability. This lower dispersion can lead to a decreased interaction with soil organisms, limited to hotspots, thus potentially lowering their toxicity.

For the actual physiochemical nanoparticle characteristics, the symbolic regression showed that the most important combination of nanoparticle descriptors (mindful that these are proxies for other descriptors) related to toxicity are *d*_BET_ and SSA and force vector of Me atoms in the core (BET size). This indicates obviously that the size (and related surface area) of the particles and the stability of the core are important for toxicity. For both 21- and 49-days exposure there was a positive relationship between core stability and presence of organisms, indicating nanoparticle-specific effects. This was most important for the 49-day exposure. In the previous work with Fe-doped TiO_2_ NPs (Fe dopped TiO_2_ nanoparticles^15^), we found that the surface force vectors were the most important, and this was related to oxidative stress since the surface forces were related to the band gap. For the Fe-doped CuO NMs, the diameter and surface area of the particles were almost in all cases positively correlated with the presence of animals at the 49-day exposure, that is, with larger particles (or with larger surface area) showing less toxicity within the size range. This was not the case for the 21-day exposure where the surface area was negatively correlated with the presence of animals.

## Conclusions

The first conclusion is that it is important to separate experimental related parameters (such as zeta potential etc.) from particle-specific descriptors (e.g., force vectors) for the best identification of important parameter/descriptors. We observed that concentration-dependent parameters (environmental parameters, e.g., zeta potential) were the most important under standard test duration (21 days); this is not the case when longer exposure studies are made (closer representing real-world conditions). In the longer exposure conditions (49 days), the particle-specific descriptors are more important than concentration-dependent parameters (usually measured under surrogate conditions, i.e., not directly in the test media). The longer-term (and more realistic) exposure showed that the steepness of the concentration–response decreased with the increased Fe content in the particles. Hence, longer-term exposure should be a requirement in the hazard assessment of NMs in addition to the previous requirement in standard OECD guidelines for chemicals.

## Materials and Methods

### Test Species

The test species *Enchytraeus crypticus* (Oligochaeta: Enchytraeidae) was used. The cultures were kept in agar, consisting of sterilized bacti-agar medium (Oxoid, Agar No. 1) and a mixture of four different salt solutions at the final concentrations of 2 mM CaCl_2_·2H_2_O, 1 mM MgSO_4_, 0.08 mM KCl, and 0.75 mM NaHCO_3_ under controlled conditions of temperature (19 ± 1 °C) and photoperiod (16:8 h light:dark). The cultures were fed with ground autoclaved oats twice per week.

### Test Materials and Characterization

A library containing homologous pure CuO and 1–10% Fe-doped CuO NMs were produced using flame spray pyrolysis as described in ref (27), and commercially available copper(II) chloride dihydrate (CuCl_2_·2H_2_O > 99.9%, Sigma-Aldrich) was used.

Briefly, the required amount of copper(II) naphthenate in mineral spirits (Strem, 8% Cu, CAS 1338-02-9) was dissolved in xylene (Strem Chemicals, 99.95% pure, CAS 1330-20-7) to obtain a 0.5 M solution by metal. To dope Fe in CuO, the required amount of iron naphthenate (12% Fe by metal, Strem Chemicals, 99.9% pure, CAS 1338-14-3) was mixed with copper naphthenate before combustion to prepare the spray solution with 1, 2, 4, 6, 8, and 10% Fe in copper naphthenate dissolved in xylene. The morphology and structural properties of the NMs produced were determined by XRD and Rietveld analysis, BET, EDX, and TEM. Further, the hydrodynamic diameter and zeta potential of the NMs were determined by DLS. The main characteristics of the NMs tested is provided in Table 1, as from Naatz et al.^27^

### Material Characterization–Modeling

The particles were characterized by atomistic modeling, as described by Tämm et al.^39^ and Burk et al.,^40^ by generating quantitative (nano)descriptors for each material. The calculations were carried out using Lennard-Jones potential^41,42^ version of the conjugate gradient approach. The core and shell region of the NP was determined by the Kneedle method,^43,44^ where it is assumed that the shell region starts where the change in the corresponding value is the highest. Consequently, the descriptors of the core atoms are quite similar to the ones that could be obtained for a perfect crystal structure. The descriptors were calculated solely based on the atomistic structure of the nanoparticle and included atom counts, core/shell distribution of atoms, coordination distances, lattice energies, etc. (see Table S1).

### Test Soil and Spiking

The standard LUFA 2.2 natural soil (Speyer, Germany) was used. The main characteristics are pH (0.01 M CaCl_2_) of 5.5, 1.77% organic matter, 10.1 mequiv/100 g of cation exchange capacity, 44.8% WHC (water holding capacity), 7.3% clay, 13.8% silt, and 78.9% sand regarding grain size distribution.

The tested concentrations were 0, 100, 320, 640, 1000, and 3200 mg NM/kg soil dry weight (DW), for the NMs, and 0, 100, 320, 640, and 1000 mg Cu/kg soil DW for CuCl_2_.

All NMs were directly mixed with the dried soil following the recommendation for dry powder nondispersible nanomaterials,^45^ done per individual replicate to ensure total raw amounts per replicate. For CuCl_2_, a stock (aqueous) solution was prepared and serially diluted and added to batches of premoisestened soil (per concentration), and the soil was homogeneously mixed. Soil moisture was adjusted to 50% of soil’s maximum WHC on adding deionized water. The soil was left to equilibrate for 24 h prior the start of the tests.

### Exposure Procedures: Survival and Reproduction

The standard guideline for the ERT^29^ and the method described by Ribeiro et al.,^46^ i.e., including an additional 28 days of exposure besides the standard 21 days (21 and 49 days), was followed, The endpoints were survival (day 21) and reproduction (days 21 and 49). Due to the limited amount of NMs available, one replicate was performed per test condition (concentration and time), except for CuCl_2_. Briefly, 10 mature adults (with well-developed clitellum) were introduced into each test container with moist soil (⌀4 cm with 20 g of soil for day 21, and ⌀5.5 cm with 40 g of soil for day 49) and food supply (24 ± 2 mg, autoclaved rolled oats). Test ran for 49 days at 20 ± 1 °C and 16:8 h photoperiod. Food (12 ± 1 mg until day 21 and 24 ± 2 mg from 21 to 49 days) and water were replenished every week. On day 21, survival and reproduction were assessed by counting the juveniles and adults. After 24 h, soil samples were sieved through meshes with decreasing pore size (1.6, 0.5, and 0.3 mm) to separate the enchytraeids from most of the soil and facilitate counting. Adult and juvenile organisms were counted using a stereomicroscope, and survival and reproduction were assessed. For the 49-day exposure replicates, on day 21, adults were carefully removed from the soil, after which the exposure continued until day 49 when the organisms were counted following procedures as described for day 21.

### Data Analysis

#### Concentration Response Modeling

Univariate effect concentrations (ECx) were calculated for each NM by modeling data to logistic or threshold sigmoid 2 parameter regression models, as indicated in Table S2 using the Toxicity Relationship Analysis Program (TRAP 1.30) software.

#### ML Analysis

To derive across material information, our data analysis protocol starts with an initial set of *N* = 68 variables *x*_1_, *x*_2_,..., *x*_68_ obtained from both computational and experimental characterization of Fe-doped CuO NMs (Table S8, BET model), being potential descriptors of the biological end point (*y*). The protocol aims to gradually prune redundant or less significant variables related to the biological response observed in the experiments, eventually identifying a limited yet essential set of descriptors and to see which computational descriptors could be used to derived toxicity models.^47^ The biological and chemical complexity along with the abundance of variables may lead to model overfitting, which we carefully considered.^48^ Similar to our previous work,^15^ the employed data analysis protocol comprises five successive steps and employs a combination of statistical and ML approaches: (i) preprocessing data, (ii) normalizing the biological response to a concentration-independent quantity, (iii) removing correlated variables, (iv) identifying descriptors through an iterative pruning process, and (v) correlating descriptors with the biological response.

The biological response to Fe-doped CuO NMs was assessed in vivo after 21 and 49 days, resulting in 40 unique biological data points (average value and standard deviation were computed in case of replicated data). The experimental characterization provided values related to concentration, material (e.g., size, chemical composition), and surrounding environment (e.g., zeta potential), along with additional variables computed by numerical modeling. Consequently, we had a 40 × 68 data matrix comprising 40 experimental results described by 68 variables (concentration, material, environment, and modeling) and 2 biological responses obtained after 21- and 49-days exposure.

First, we cleaned the initial data set by removing variables with missing data. Second, we fitted the biological response (*y*) using a logarithmic equation (*y* = −*b* ln *c + b*_0_) as a function of nanoparticle concentration (*c*) for each of the materials in the library. This provided us with an end point that integrated the entire concentration response curve. This concentration-independent end point (*b*) directly integrates the biological response over all exposures and allows for a clear identification of the NM descriptor parameters responsible for the toxicity. Third, we pruned the data by identifying and clustering redundant variables (highly correlating variables) to achieve a shorter list of noncorrelating variables. We used the hierarchical clustering algorithm with the Spearman’s correlation coefficient as the metric to quantify similarity between variables.^49^ Variables with similar characteristics were hierarchically linked and grouped into clusters until the stopping criteria were met (here, the inconsistency coefficient equal to 0.8, roughly corresponding to the 1-sigma confidence level). A representative variable per cluster was nominated with preference given to variables commonly studied in the toxicity literature. However, it is essential to note that for our specific purposes, any variable within the cluster holds equal significance. Fourth, we iteratively pruned the uncorrelated concentration-independent variables obtained from the clustering step to identify the most significant descriptors for the normalized biological response after 49-days exposure. At each pruning step, a symbolic regression algorithm was used to find accurate and compact functions (*f*) relating the available *N*_*i*_ variables (*x*_1_, *x*_2_,..., *x*_*Ni*_) to the concentration-independent end point (*b*). The complexity of these *f* functions was compared with the resulting fitting accuracy using a Pareto front approach (e.g., Figure S1). At each *i*th pruning step, we selected the best ranked 40% of variables based on their occurrence in suitable *f* functions on the Pareto front and pruned the remaining ones, therefore obtaining a reduced set of *N*_*i+1*_ variables to be analyzed in the successive (*i* + 1)th pruning step. This process was iteratively performed until a stopping criterion was reached, defined as the ratio between the average and standard deviation of the weighted importance for the remaining variables being less than 0.2. Higher values of this ratio indicate the presence of variables with limited effect on the concentration-independent end point, which should be further pruned from the analysis. The symbolic regression algorithm implemented in Eureqa software was used, employing different parametrizations of the minimization algorithm and averaging fitting results. In more detail, we considered two sets of building blocks for the explored fitting equations: rational polynomial functions and a combination of rational polynomial, exponential/logarithmic, and square root functions. Additionally, we employed three target error metrics: maximizing the R-square, minimizing the absolute error, and maximizing a hybrid correlation/error index. This resulted in six different repetitions of the fitting procedure for each pruning step. A stable solution was typically reached after 2–20 million generations. To aid convergence of the minimization algorithm, the data were preliminarily normalized with respect to their mean and standard deviation per each independent/dependent variable. Fifth, the concentration-independent variables that remained after the pruning process were considered relevant descriptors related to the biological response to the tested Fe-doped CuO NMs, along with concentration-dependent parameters such as the concentration, hydrodynamic size, and zeta potential. Finally, we used the symbolic regressor to best fit the biological response (*y*) with the remaining descriptors, refining the minimization process through more than 40 million iterations. Based on this final fitting procedure, we assessed the sensitivity between each descriptor and the biological response for both 21- and 49-days exposure. Further information on the methodological details of the ML analysis is reported in our previous work.^15^

## Supporting Information Available

Pareto front list of suitable fitting functions *f* identified by the symbolic regressor; Spearman’s correlation coefficient computed between each pair of Fe-doped CuO particles variables potentially related to toxicity; Spearman’s correlation coefficient computed between each pair of concentration-independent variables within the 15 clusters identified by the hierarchical clustering algorithm; results of variable pruning; and comparison of model correlations between the biological responses (PDF)
Details of the variables used for ML modeling; characteristics of the Fe-doped CuO particle library, including atomistic modeling; effect concentrations (ECx) estimated; best fitting parameters and corresponding *R*^2^ determined for the Fe-doped CuO particles assessed in vivo (21 days); best fitting parameters and corresponding *R*^2^ determined for the Fe-doped CuO particles assessed in vivo (49 days); clustering of correlated concentration-independent variables; uncorrelated concentration-independent variables representative of each cluster considered in the pruning process; uncorrelated descriptors of the biological response y for the tested Fe-doped CuO particles; and curated data set considered for the ML analysis of Fe–Cu oxide nanoparticles (XLSX)
