# Supplementary material for: Machine Learning Allowed Interpreting Toxicity of a Fe-Doped CuO NM Library Large Data Set—An Environmental In Vivo Case Study
Source: ACS Appl Mater Interfaces. 2024 Aug 1;16(32):42862–72. doi: 10.1021/acsami.4c07153 (PMC11331442; doi:10.1021/acsami.4c07153)
Supplement: Supplementary file 1 — am4c07153_si_001.pdf [file am4c07153_si_001.pdf]

Suporting Information

Machine Learning Allowed Interpreting Toxicity of a Fe Doped-CuO NMs Library Large Dataset – an Environmental in Vivo Case Study

Janeck J. Scott-Fordsmand[a], Susana I.L. Gomes[b], Suman Pokhrel[c,d], Lutz Mädler[c,d], Matteo Fasano[e], Pietro Asinari[e,f], Kaido Tämm[g], Jaak Jänes[g] and Mónica J.B. Amorim[b*]

[a]Department of Ecoscience, Aarhus University, C.F. Møllers Alle 4, DK-8000, Aarhus, Denmark

[b]Department of Biology & CESAM, University of Aveiro, 3810-193 Aveiro, Portugal

[c]Department of Production Engineering, University of Bremen, Badgasteiner Str. 1, 28359 Bremen, Germany

[d]Leibniz Institute for Materials Engineering IWT, Badgasteiner Str. 3, 28359 Bremen, Germany

[e]Department of Energy, Politecnico di Torino, Corso Duca degli Abruzzi 24, Torino 10129, Italy

[f]INRIM, Istituto Nazionale di Ricerca Metrologica, Strada delle Cacce 91, Torino 10135, Italy

[g]Institute of Chemistry, University of Tartu, Ravila 14a, Tartu 50411, Estonia

*Corresponding author:

mjamorim@ua.pt;

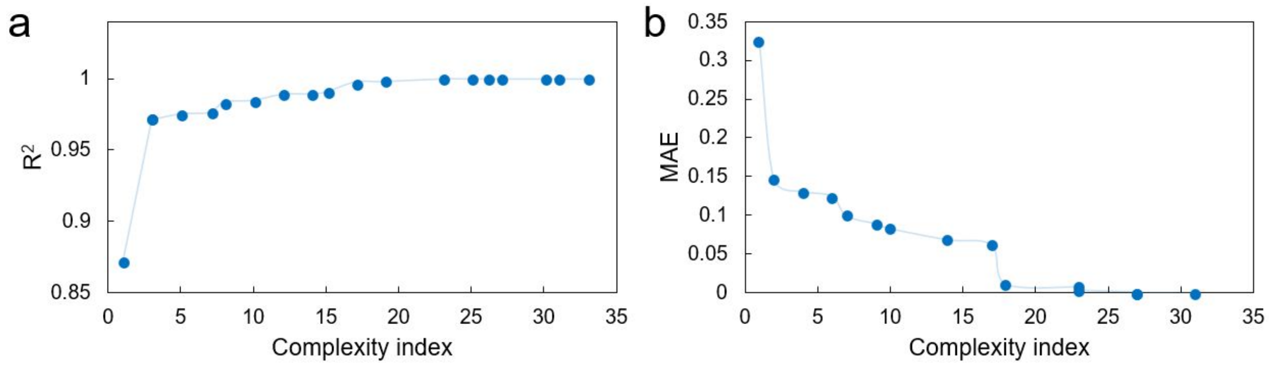

Figure S1. The Pareto front represents a list of suitable fitting functions *f* identified by the symbolic regressor, showing the trade-off between two key metrics: (a) the increase in R^2, indicating higher accuracy with more complex equations, and (b) the decrease in Mean Absolute Error (MAE). The most complex fitting equation tends to be the most accurate, while the elbow of the Pareto front signifies the best balance between fitting accuracy and equation complexity. To quantify the complexity of equations, the Eureqa symbolic regressor assigns default scores to formula building-blocks: 1 for constant, addition, subtraction, and multiplication; 2 for division; and 4 for exponential, natural logarithm, and square root functions. The results depicted in this figure refer to one repetition of the 1st pruning round of concentration-independent variables.

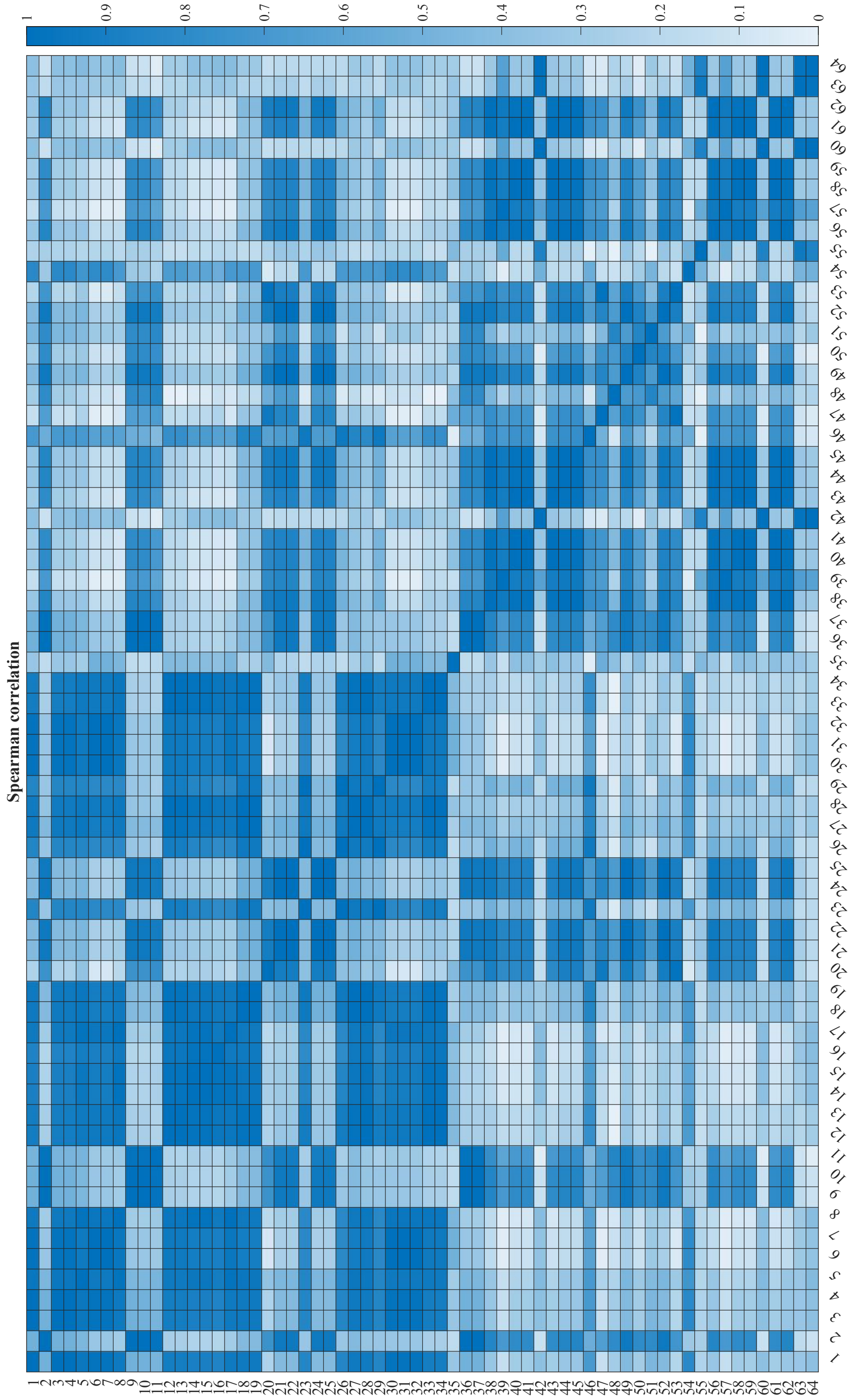

Figure S2. Spearman's correlation coefficient computed between each pair of Fe-doped CuO particles variables potentially related to toxicity. In detail, the figure displays the 64 concentration-independent variables that remained after dataset cleaning. Whiter colour tones in the figure indicate no correlation between the variables, while blue tones indicate correlation. It is important to note that, as per the definition of Spearman's correlation coefficient, the matrix is symmetrical. See **Table S8** for a detailed description of the 64 concentration-independent variables considered in this analysis.

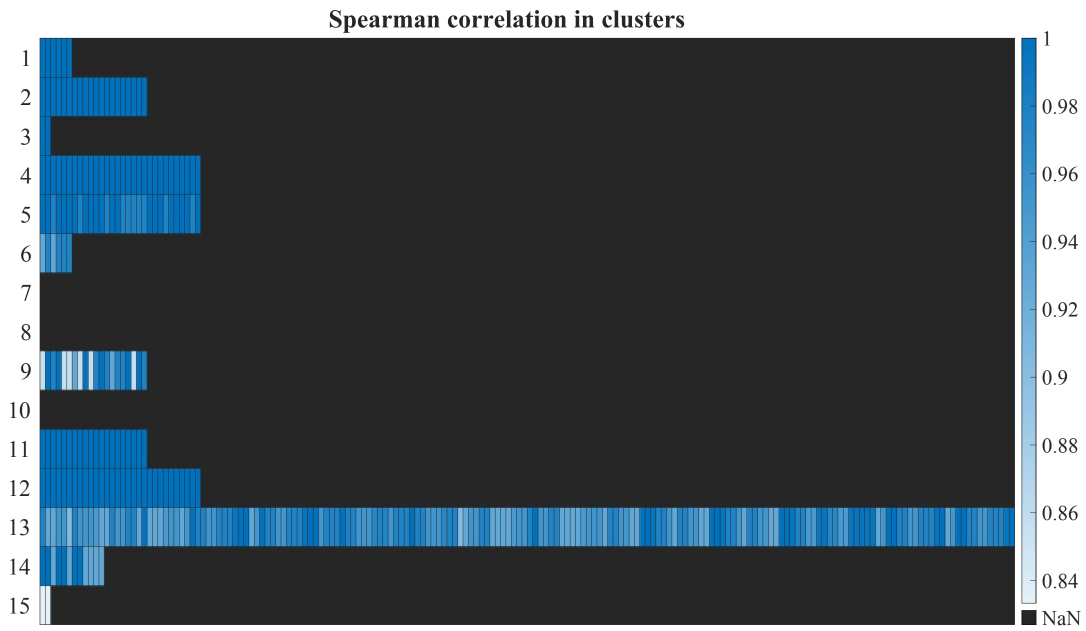

Figure S3. Spearman's correlation coefficient computed between each pair of concentration-independent variables within the 15 clusters identified by the hierarchical clustering algorithm (refer to **Table S5**). In the figure, whiter colour tones indicate less correlation between each pair of variables, while blue tones indicate higher correlation. The black colour represents the background of the figure. It is important to note that the Spearman's correlation coefficient cannot be computed within clusters consisting of only one variable (e.g., cluster #7).

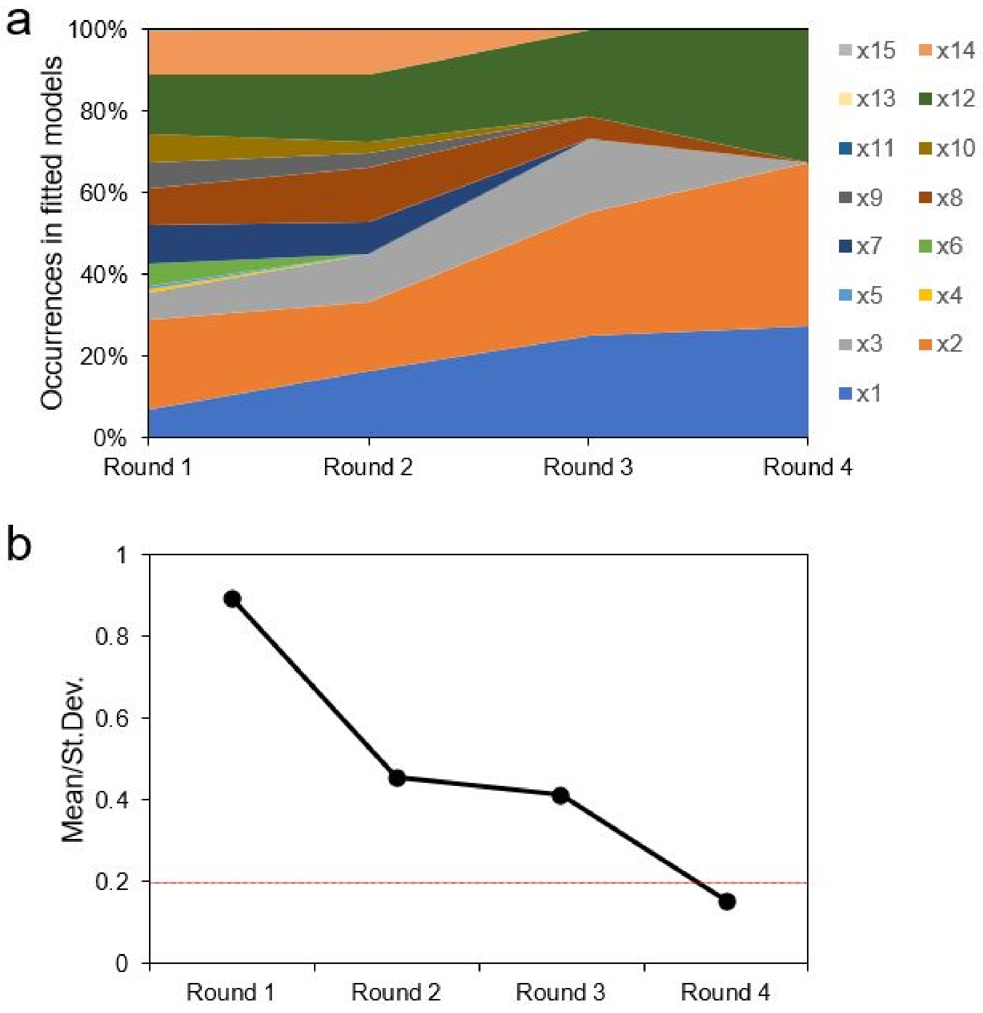

Figure S4. Results of variables pruning. **(a)** Normalized occurrences of concentration-independent variables x_i in the fitting functions f identified by the symbolic regressor for the concentration-independent endpoint b. The definitions of the reported variables x_1, …, x_{15} are reported in the **Table S6**. Several rounds of pruning are carried out, in which only the best ranked 40% of variables in terms of occurrence are kept, while the remaining ones pruned. **(b)** This process is repeated until the considered stopping criterion (i.e., the ratio between average and standard deviation of normalised occurrences of concentration-independent variables in the fitting functions f) is met – see the horizontal red line. This is achieved at the 4th pruning round.

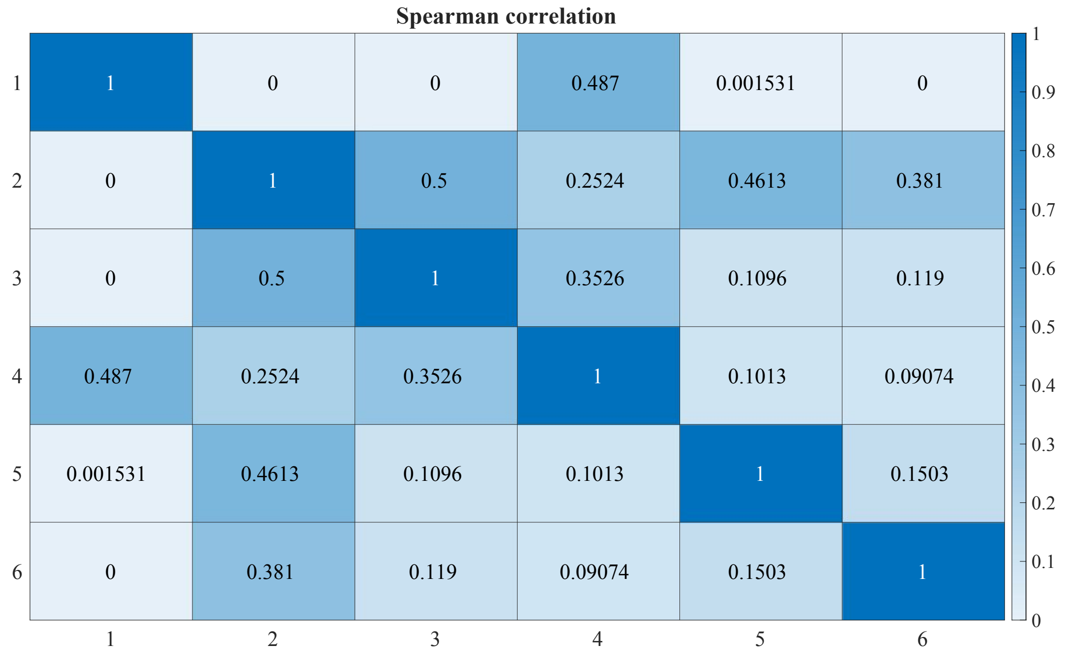

Figure S5. Spearman's correlation coefficient computed between each pair of Fe-doped CuO particles descriptors related to toxicity. In detail, the figure displays the 3 concentration-independent descriptors that remained after the pruning process and the 3 concentration-dependent descriptors, as detailed in **Table S7**. Whiter colour tones in the figure indicate no correlation between the variables, while blue tones indicate correlation. It is important to note that, as per the definition of Spearman's correlation coefficient, the matrix is symmetrical. Results show that the reported descriptors are uncorrelated between each other.

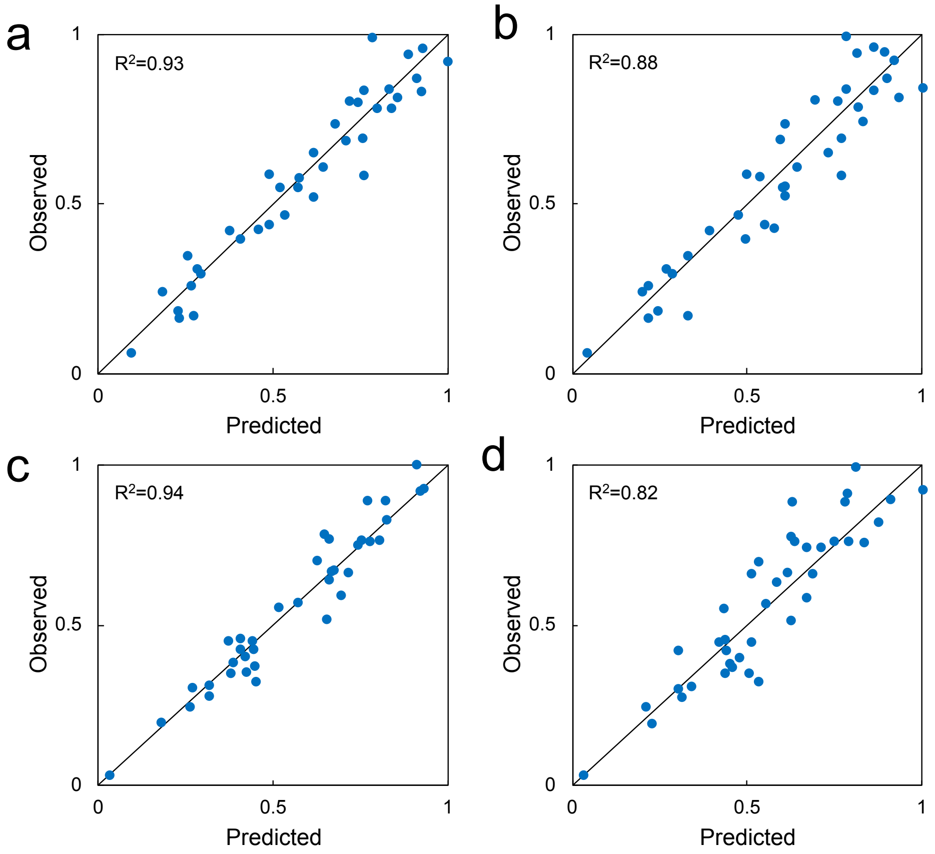

Figure S6. Comparison of model correlations between the biological response (y) and the identified descriptors (x_1, ..., x_6, see **Table S7** for details) for Fe-doped CuO particles: experimental observations vs. model predictions. The values are normalized using the min-max approach, and each dot represents one tested configuration. **(a)** Fitting performance of the most complex and accurate function for Fe-doped CuO particles after 21 days exposure. **(b)** Fitting performance of the best compromise between model complexity and accuracy (i.e., the elbow of the Pareto front) for Fe-doped CuO particles after 21 days exposure. **(c)** Fitting performance of the most complex and accurate function for Fe-doped CuO particles after 49 days exposure. **(d)** Fitting performance of the best compromise between model complexity and accuracy for Fe-doped CuO particles after 49 days exposure.
